# Current European *Labyrinthula zosterae* Are Not Virulent and Modulate Seagrass (*Zostera marina*) Defense Gene Expression

**DOI:** 10.1371/journal.pone.0092448

**Published:** 2014-04-01

**Authors:** Janina Brakel, Franziska Julie Werner, Verena Tams, Thorsten B. H. Reusch, Anna-Christina Bockelmann

**Affiliations:** 1 Experimental Ecology – Food webs, Geomar Helmholtz Centre for Ocean Research Kiel, Kiel, Germany; 2 Evolutionary Ecology of Marine Fishes, Geomar Helmholtz Centre for Ocean Research Kiel, Germany; University of Heidelberg, Germany

## Abstract

Pro- and eukaryotic microbes associated with multi-cellular organisms are receiving increasing attention as a driving factor in ecosystems. Endophytes in plants can change host performance by altering nutrient uptake, secondary metabolite production or defense mechanisms. Recent studies detected widespread prevalence of *Labyrinthula zosterae* in European *Zostera marina* meadows, a protist that allegedly caused a massive amphi-Atlantic seagrass die-off event in the 1930's, while showing only limited virulence today. As a limiting factor for pathogenicity, we investigated genotype×genotype interactions of host and pathogen from different regions (10–100 km-scale) through reciprocal infection. Although the endophyte rapidly infected *Z. marina*, we found little evidence that *Z. marina* was negatively impacted by *L. zosterae*. Instead *Z. marina* showed enhanced leaf growth and kept endophyte abundance low. Moreover, we found almost no interaction of protist×eelgrass-origin on different parameters of *L. zosterae* virulence/*Z. marina* performance, and also no increase in mortality after experimental infection. In a target gene approach, we identified a significant down-regulation in the expression of 6/11 genes from the defense cascade of *Z. marina* after real-time quantitative PCR, revealing strong immune modulation of the host's defense by a potential parasite for the first time in a marine plant. Nevertheless, one gene involved in phenol synthesis was strongly up-regulated, indicating that *Z. marina* plants were probably able to control the level of infection. There was no change in expression in a general stress indicator gene (HSP70). Mean *L. zosterae* abundances decreased below 10% after 16 days of experimental runtime. We conclude that under non-stress conditions *L. zosterae* infection in the study region is not associated with substantial virulence.

## Introduction

In the recent past, microorganisms, associated with multi-cellular organisms, have been receiving increasing attention as a driving factor in ecosystems (e.g. [1]). Endophytes in plants can change host growth and shoot production [2] by altering nutrient uptake [3], secondary metabolite production or defense mechanisms [4]. Moreover, endophytes can be parasites and thereby play a crucial role in ecosystems by controlling the dynamics of host populations, by regulating host abundances and, thus, by contributing to ecosystem stability [5]. In the marine realm, emerging diseases caused by microorganisms, have been recognized as causes for species extinction, regime shifts or altered community structure [6], [7]. How two species interact, whether the host benefits or is degraded by the microbe depends mainly on two factors: the effectiveness of the defense reaction of the host and the pathogenicity of the microorganism.

In this study we investigated the interaction of the most abundant seagrass in the northern hemisphere [8], *Zostera marina*, with the endophytic protist *Labyrinthula zosterae*, which caused the world's largest reported seagrass die-off event. Seagrasses form one of the most valuable coastal ecosystems on earth [9]. They are marine flowering plants, which form huge meadows, providing food, shelter and settlement substrate for many organisms. Being the foundation species of one of the most productive ecosystems [10], they sequester 15% of the total marine consumed CO_2_ and represent thereby an important sink and storage of atmospheric CO_2_ [11]. Seagrass meadows contribute to coastal protection [12], play a key role in nutrient cycling [13] and add to water clarity by reducing current velocity and by increasing sedimentation [14]. Seagrasses are sensitive to reduced light availability due to eutrophication [15] or increasing water turbidity [16]. Since anthropogenic impact on this sensitive ecosystem is still increasing, seagrass populations are declining worldwide [16], [17].

In the 1930's, the so called ‘wasting disease’ affected *Z. marina* populations along the Atlantic coasts of North America, the European Atlantic, the North and Wadden Sea and the Baltic Sea, affecting eelgrass populations in France, Great Britain, The Netherlands, Germany and Denmark (for review see [18], ). During the ‘wasting disease’ epidemic more than 90% of the Atlantic coast eelgrass populations disappeared [19] after repeatedly developing expanding black or brown lesions on the leaf blades that finally resulted in a disintegration of the rhizome and death of the plants. The eelgrass loss had a tremendous impact on the eelgrass associated fauna (reviewed by [19]). Recovery of the *Z. marina* populations was slow [21] and in some areas eelgrass never recovered, e.g. the western Wadden Sea [22]. In the 1980's, a reoccurrence of the ‘wasting disease’ was reported from New Hampshire and Maine [21], [23], [24].

Already in the 1930's, Renn [25] proposed a marine slime mold, *Labyrinthula sp.*, as the agent of the ‘wasting disease’. In 1988 Muehlstein *et al.* [26] confirmed, by applying Koch's postulate, *Labyrinthula zosterae* to be the causative agent of the wasting disease.

Recent studies detected widespread prevalence of the protist *Labyrinthula zosterae* in European eelgrass (*Zostera marina*) meadows [27], demonstrating that *L. zosterae* is still an integral part of the eelgrass ecosystem. The *L. zosterae*-strains currently occurring in northern European eelgrass meadows apparently cause neither massive disease symptoms nor die-offs. The primary objective of this study was to better understand the *Z.marina* – *L. zosterae* interaction, by gaining information about the host's defense mechanisms as well as local co-adaptations of both, host and microbe. This insight may also enable us to explain the actual absence of the disease and to predict the risk of future lethal epidemics in seagrass beds.

**Figure 1:**
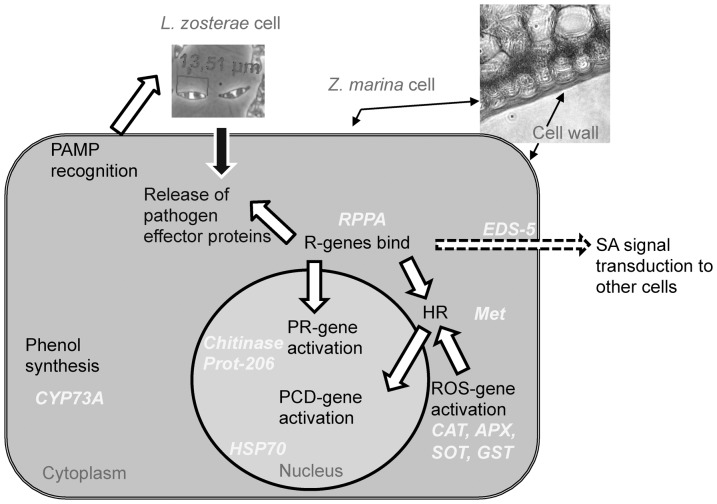
Defense mechanism of *Zostera marina*.

Nothing is known about pathogen defense in *Z. marina* specifically, but in general, flowering plant defense reactions against pathogens are evolutionary conserved [28] and can be understood as a cascade with different layers (Fig. 1). First, physical (e.g. wax cuticle or cell walls) and biochemical barriers (e.g. antimicrobial enzymes or secondary metabolites) inhibit pathogen growth [29]. One important group of secondary metabolites are phenolic acids and their derivates, which have various functions, for examples antioxidant capacity [30] and antimicrobial function [31]. Accumulation of phenolic compounds probably also plays a role in the interaction between *Z. marina* and *L. zosterae*, since higher concentrations of phenolic acids, mainly caffeic acid, were detected in infected as compared to healthy plants [32].

Secondly, receptors at the cell surface recognize slow evolving pathogen (or microbe) associated molecular patterns (PAMPs = MAMPs, e.g. bacterial flagellin or fungal chitin), which induce a basal defense [33]. However, some pathogens can overcome this defense induction by inhibiting the pathway through release of effector proteins into the host tissue. As a counter response, most plants demonstrate cytoplasmic or membrane-localized receptors (so called resistance-genes or R-genes), that bind directly to pathogen-released effectors or to damaged host cell fragments [34]. Upon binding to the receptor, reactions are triggered that can induce a hypersensitive response (HR) and the expression of a set of pathogenesis-related proteins [35]. HR is mediated by metacaspases and other factors, such as hydrogen peroxide concentration. In HR, the infected cell undergoes a programmed cell death (PCD or apoptosis), which limits the reproduction and spread of the pathogen within the host tissue [36]. As a final level of defense, pathogenesis-related genes (PR-genes) are expressed such as chitinases, defensins or beta-1,3-glucanase, which work against pathogens in various ways [37].

During induction and regulation of plant defense reactions, plant hormones spread information about infection throughout the plant, which might lead to systemic resistance. In general, Salicylic acid (SA) seems to be the dominant hormone in biotrophic pathogen interaction, while Jasmonicacid (JA) and Ethylene (ET) have been found to be involved more frequently in necrotic interaction [38].

In regard to the lack of virulence of today's *L. zosterae* infection, several explanations are possible. First, the genotypes of the protist currently present may generally show low or no virulence. This was tested by experimentally inoculating naïve *Z. marina* raised from seeds with *L. zosterae*. Second, plant genotypes may be adapted to local protist genotypes (in particular in historical wasting disease areas) preventing virulence effects. Hence, we investigated the host – pathogen co-adaptation in different populations on a regional spatial scale by applying a reciprocal infection design to test infectiousness and pathogenicity. Third, we characterized the defense reaction of *Z. marina* after infection with *L. zosterae* by measuring the gene expression of 11 defense related genes that were identified using *Z. marina* EST library sequences [39] via comparison of gene models of terrestrial model plants at different time intervals post infection. We choose genes from different levels of the defense cascade (Fig. 1). We aimed to answer the following research questions:

1. How virulent is *Labyrinthula zosterae* in the study area (measured as lesion development, leaf growth and leaf production by *Zostera marina*; Experiment I: experimental inoculation of the eelgrass hosts with *L. zosterae*)?
2. Are there differences in infectiousness and virulence between *Zostera marina* hosts and *Labyrinthula zosterae* endophytes with different origin, which may explain local persistence of host and pathogen (Experiment I: Reciprocal inoculation of eelgrass hosts and endophyte with *L. zosterae*, both with different origin)?
3. Does infection of *Zostera marina* by *Labyrinthula zosterae* lead to enhanced expression of defense related genes (Experiment II: Defense gene expression in *Zostera marina*)?

## Materials and Methods

### Seed collection, germination and cultivation of *Zostera marina*

In order to raise *L. zosterae* naïve plants for experiment I, we collected about 100 flowering shoots with seeds from each of three subtidal populations along the north-western German Baltic (Wackerballig in Flensburg Fjord, Kiekut in Eckernförde Bay and Strande in Kiel Fjord) in July 2010 (Table 1). No specific permissions were required for these locations/activities, since GEOMAR research activities along the coasts and shelf areas in the Baltic Sea are permitted when adhering to the general guidelines for the operation of research vessels. Our field studies did not involve endangered or protected species. In October 2010, another 100 flowering shoots were collected from a subtidal population of *Zostera marina* in List on the island of Sylt in the German Wadden Sea (Table 1). Sampling at Ellenbogen Creek was permitted by the nature conservation authority and Mr. Diedrichsen, the owner of this private property. Collected flowering shoots were immediately transported in water containers to GEOMAR Kiel and stored floating in mesocosms, in filtered seawater at 21°C and with the respective sampling site's salinity until seeds were ripe.

**Table 1:** Sampling sites of *Zostera marina*.

Area	Location	Geograph. Coordinates	Sampling date	Salinity (psu)	Sampled
**Experiment 1**					
Sylt, Wadden Sea,	List	N 55.0410	October 2010	>30	Flowering shoots, leaves
Germany		E 08.4130	August 2011		for isolation of *L. zosterae*
Flensburg Fjprd,	Wackerballig*	N 54.7557	July 2010	15–17	Flowering shoots, leaves
Germany		E 09.8668	August 2011		for isolation of *L. zosterae*
Eckernförde Bay,	Kiekut	N 54.4483	July 2010	15–17	Flowering shoots, leaves
Germany		E 08.7106	August 2011		for isolation of *L. zosterae*
Kiel Fjord,	Strande	N 54.4330	July 2010	15–17	Flowering shoots
Germany		E 10.1699			
Kiel Fjord,	Falckenstein	N 54.3954	August 2011	15–17	Leaves for isolation of *L.*
Germany		E 10.1935			*zosterae*
**Experiment II**					
Kiel Fjord,	Strande	N 54.4330	June 2011	15–17	Flowering shoots, leaves
Germany		E 10.1699	July 2012		for isolation of *L. zosterae*

*Leaves for isolation of *L. zosterae* were harvested from plants infected in experiment I and kept in mesocosms until March 2012.

Ripe seeds were stored at 5°C for stratification (September–November 2010: Baltic seeds; November 2010–January 2011: Wadden Sea seeds). Subsequently, *Zostera marina* seeds were sown in plastic aquaria filled with ambient sediment and submerged in mesocosms with ambient sea water (15 psu) at 10°–12°C and with 12 hours light (∼600 µE m^−2^ s^−1^).

When seedlings reached a size of 10–15 cm in March–April 2011, 6 seedlings were transferred to each plastic aquarium holding sediment of 25 cm thickness, submerged in 50×50×100 cm aerated containers with a 1∶1 mixture of Kiel Fjord Sea and North Sea water (25 psu). Each seedling received ∼0.02 g Nitrate and ∼0.009 g Phosphate (Plantacote Mix 4M, Manna, Germany). Temperature was raised to 17°C and a light∶ dark regime of 15 ∶ 9 was applied to mimic early summer conditions. One third of the water was exchanged every week.

*Zostera marina* seeds for experiment II were collected in an eelgrass population close to Strande (Table 1) in June 2011. No specific permissions were required for these locations/activities (see above). The procedure was identical to the first experiment. Seeds germinated between December 2011 and February 2012. In March 2012, *Z. marina* seedlings were planted into aquaria. Temperatures were continuously increased from 12°C in March to 18°C in August. The light period was extended from 12 hours in March to 16 hours in August.

### *Labyrinthula zosterae* isolation and cultivation

For isolation of *L. zosterae* for experiment I, we sampled leaves from vegetative *Zostera marina* shoots at the seed sampling sites List, Kiekut and Falckenstein. *Labyrinthula zosterae* was isolated and cultured on seawater-agar-medium as previously described [18]. In preparation of the infection procedure, we autoclaved medical gauze compresses (Lohman und Rauscher, Germany). Five squares of gauze (1.5×1.5 cm) were placed in a circle on each seawatermedium plate. We then inoculated the centre of these plates with *L. zosterae* cells, resulting in an identical distance of all gauze pieces to the inoculated *L. zosterae* culture. After 5 days the gauzes were overgrown by *L. zosterae*. Four different strains of *L. zosterae* were used for each original site (see below). *L. zosterae* DNA from one gauze piece of each culture was extracted (see below) and subjected to real-time quantitative PCR analysis (rt-QPCR, see below) for the determination of inoculation concentration of *L. zosterae*. Inoculation concentration was 15,310±3,240 *L. zosterae* cells/square of gauze.

In experiment II the isolation of *L. zosterae* cultures for infection was identical to experiment I. Here, we sampled *Z. marina* leaves from Strande (Table 1) in July 2012 and received three different *L. zosterae* strains. The gauze bandages used for inoculation were rectangular and smaller (1.5×0.75 cm, 6,017±853 *L. zosterae* cells/square of gauze) in this case.

### Experiment I: Reciprocal infection of host and endophyte with different origin

#### Experimental design

**Figure 2:**
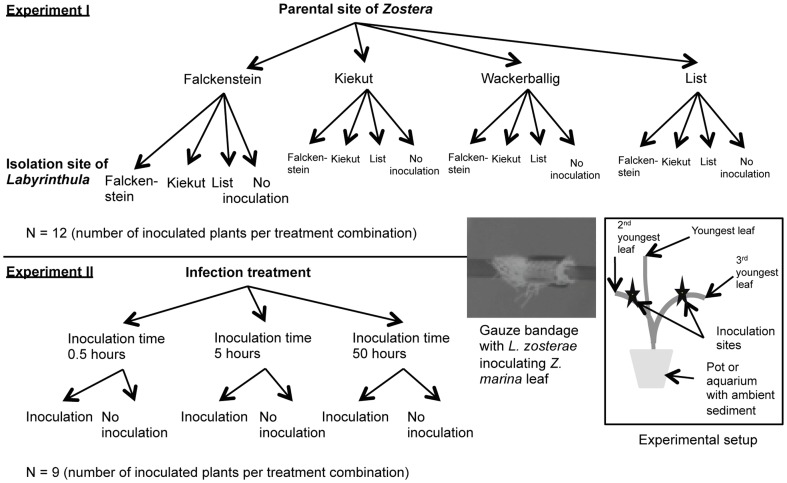
Experimental design and setup of experiment I and II.

Before the start of the experiment on August 25^th^, 2011, 48 plastic aquaria (15×25 cm) were filled with 10 cm of ambient, sterilized sediment. Six *Zostera marina* seedlings from one of the four parental sites (experimental factor 1, Fig. 2) were planted in each aquarium, resulting in 12 aquaria per parental site. Each seedling received slow-release fertilizer (see above) again and was given six weeks for settlement. After that, one aquarium from each parental side was placed in each one of 12 mesocosms. The latter were filled with 600 L of a mixture of Kiel Fjord and North Sea water resulting in a salinity of 25 psu at a temperature of 18–19°C. During the experiment 1/3 of the water was exchanged every week and temperature and salinity were controlled every other day. The light period was 16 hours.

For infection, the second and third oldest leaf of each *Z. marina* shoot was wrapped with a gauzed bandage containing *Labyrinthula zosterae* from different isolation sites (second experimental factor, Fig. 2, Table 1) for 24 hrs. All plants in aquaria of the same mesocosm received bandages from the same isolation site, resulting in three mesocosms with four aquaria and 72 plants per isolation site. Plants in the remaining three mesocosms were not infected. The second and third oldest leaf of three of the six plants was wrapped with non-infected bandage to control for an effect of the bandage itself. After one day all bandages were removed and infection success was determined by the appearance of lesions on the leaf surface.

The size of the lesions was determined by estimating the fraction of the leaf that had turned black in five classes (0%, >0–10%, >10–25%, >25–50%, >50–75%, >75–100%). We assessed lesion size one, two, three, six and nine days after infection on the second oldest leaf. Lesions on the third oldest leaf were estimated one, two, three, six days after infection. At day three the leaf 3^rd^ was harvested and dried for *L. zosterae* determination by rt-QPCR. Furthermore, we measured leaf length of the third oldest, second oldest and youngest leaf at the start of the experiment and at day six. After harvesting the third oldest leaf, leaf length of the second oldest (as far as it was present and not naturally shed), youngest and all newly appearing leaves was measured after 10, 17 and 32 days. On day 32 after infection, the first leaf that appeared post infection was harvested and analyzed by rt-QPCR for *L. zosterae* infection.

#### DNA-extraction and real-time quantitative PCR assay (rt-QPCR)

After sampling, the harvested leaves were air dried. Approximately 2–4 mg dried leaf material from 2–3 cm above and below the region where infective gauze bandage had been placed was first ground in a ball mill (Retsch, Germany) at maximal speed (4×8 min.). DNA extractions of *L. zosterae* were performed with an Invisorb spin tissue mini kit (Invitek, Berlin, Germany) following the manufacturer's instructions. To enhance extraction efficiency and to ensure that even low amounts of target DNA were carried through the filter absorption steps, 1 µL (containing ∼500 ng) of UltraPure salmon sperm DNA solution (Invitrogen, Life Technologies, USA) was added to each extraction to saturate silica columns with DNA. Target DNA was purified using a one-step PCR inhibitor removal kit (Zymo Research, USA).

To determine *Labyrinthula zosterae* cell number, we followed a TaqMan based rt-QPCR assay as described in Bockelmann *et al.* [18] with a fluorescently-labeled ITS probe.

In one reaction we used 10 µL TaqMan universal Master Mix (Applied Biosystems, now Life Technologies) in a 20 µL reaction volume: 2 µL 1∶10 diluted template DNA, 2.4 µL (40.8 nM) of the two primers, 2.4 µL Milli-Q H_2_O and 0.8 µL probe (50 nM), respectively. The thermo-cycling program on a Step-One QPCR machine was 2 min at 50°C and 10 min at 95°C, followed by 48 cycles at 95°C for 15 s and 1 min at 60°C.

#### Data analysis and statistics

Lesion size was estimated as percent data and had to be arc sine transformed to achieve variance homogeneity.
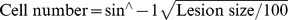



Growth rates for individual leaves were calculated as
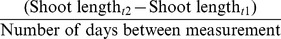
Growth rates and leaf production (number of new leaves produced post infection) data were log transformed.

All samples analyzed by rt-QPCR were tested in triplicate and the standard deviation of triplicates never exceeded 0.5 units of cycle threshold (Ct). Only CT values <39 were considered.

Standard curves using preparations of *Labyrinthula zosterae* with known cell numbers attained correlation coefficients between r^2^ = 0.97 and 0.99 and a detection limit of ∼0.01 cells. Abundance as the number of *L. zosterae* cells in each milligram (dry weight) *Zostera marina* sample was calculated from the linear regression of the standard curve (Standard cell number against mean Standard Ct calculated from all rt-QPCR reactions; 150 cells = 22.493 Ct±0.060 SE, 15 cells = 27.080 Ct±0.080 SE, 0.5cells = 32.215 Ct±0.125 SE).
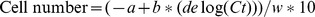
where a = intercept, b = slope and w = sample dry weight. Cell number has to be multiplied by 10 because the samples were diluted 1∶10 prior rt-QPCR.

Statistical analysis was based on a general linear model and done by 2-way analysis of variance (implemented in software JMP 9, SAS Institute, USA). “Parental site” of *Zostera marina* (Kiel Fjord, Eckernförde Bight, Flensburg Fjord and Sylt) and “Isolation site” of *Labyrinthula zosterae* (Kiel Fjord, Eckernförde Fjord, Sylt and no infection) were independent factors in the model. The control treatments were analyzed as a forth level of the factor isolation site. Dependent factors were “lesion size”, “growth rate/day”, “leaf production” and “*L. zosterae* cells/mg *Z. marina* dry weight”. Table 2 summarizes the results of the statistical analysis.

**Table 2:** Experiment 1: Statistical analysis of differences in *Labyrinthula zosterae* abundance, lesion size, growth rate and leaf production after inoculation of *Zostera marina* with *L. zosterae* compared with uninoculated plants.

Response variable	Factor	df	SS	F/χ^2^	P	Residual SS
*L. zosterae* abundance*	*Z. marina* origin	3		6.39	0.09	
	*L. zosterae* origin	3		46.47	**<0.0001**	
Lesion size leaf 3§	*Z. marina* origin	3	0.32	3.81	**0.01**	6.74
	*L. zosterae* origin	3	9.77	119.27	**<0.0001**	
	*Z.m*ori..×*L.z*. ori	9	0.28	1.15	0.33	
Lesion size leaf 2§	*Z. marina* origin	3	0.45	2.49	0.06	14.56
	*L. zosterae* origin	3	11.67	63.81	**<0.000**	
	*Z.m*ori..×*L.z*. ori	9	0.77	1.41	0.18	
Growth rate *Z.m.* leaf 2‡	Inoculated vs. not inoculated	1	0.13	0.15	0.697	106.33
Growth rate *Z.m.* leaf 1‡	Inoculated vs. not inoculated	1	1.44	5.40	**0.021**	61.70
Growth rate *Z.m.* leaf 0^3^	Inoculated vs. not inoculated	1	6.57	9.10	**0.003**	159.62
Leaves produced post infection‡	Inoculated vs. not inoculated	1	0.87	16.64	**0.0003**	15.47

* = Wilcoxon Test,

§ = lesion size 3 days post inoculation, 2-way-ANOVA,

‡ = 1-way-ANOVA.

### Experiment II: Defense gene expression in *Zostera marina*

**Figure 3:**
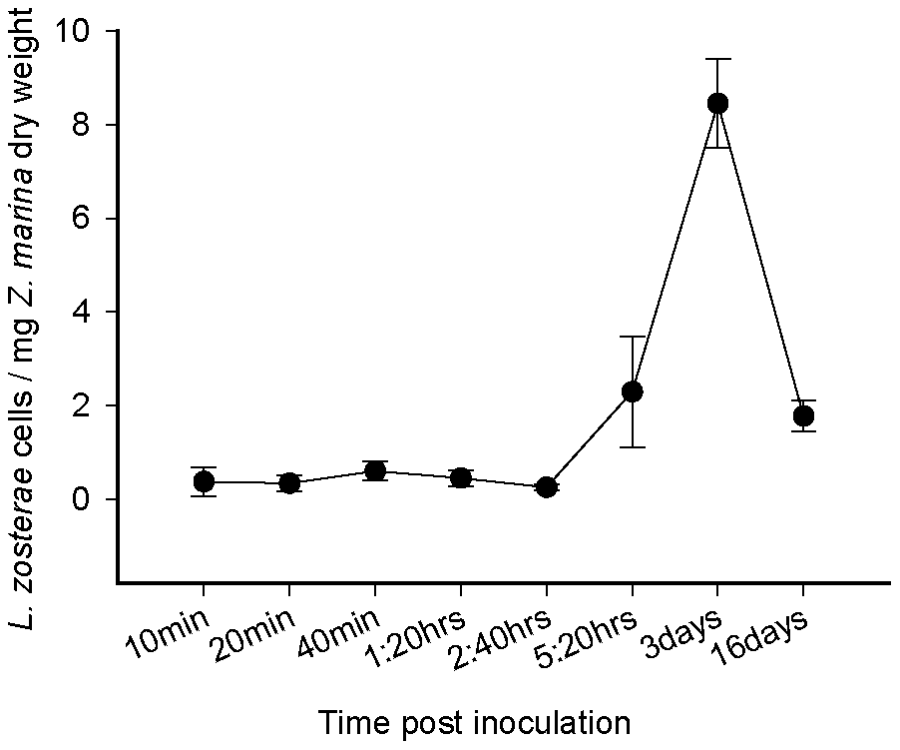
Abundance of *Labyrinthula zosterae* cells per mg *Zostera marina* leaf sample (dry weight) depending on inoculation time during experimental *L. zosterae* infection. Results are partly from experiment I and II, means with standard error bars.

The objective of the second experiment was to analyze the *Zostera marina* defense reaction in a target-gene approach. In a pilot experiment, we first tested the abundance of *L. zosterae* within *Z. marina* leaves after different inoculation times in order to investigate how much time the protist needs to enter an eelgrass leaf. *Zostera marina* and *Labyrinthula zosterae* were both collected from an eelgrass population in the Eckernförde Bay (Table 1). The plants were either cultured from seeds (see above) or sampled in February 2012, when *L. zosterae* prevalence in the population showed to be minimal [18]. *Labyrinthula zosterae* cultures were isolated from *Zostera marina* plants, which had been infected in experiment I and had been cultivated in our mesocosm facility thenceforth. On April 24^th^ and 25^th^ the 2^nd^ and 3^rd^ youngest leaves of each plant were infected and sampled. We tested incubations of 10, 20, 40, 80, 160 and 320 minutes. To control for accidental infection prior to the experimental infection treatment, we took samples from all plants before infection treatment. Cell numbers of *Labyrinthula zosterae* per mg *Zostera marina* dry weight were obtained and tested in the same way as described for experiment I (see above). This pilot study revealed that the first plants were infected after 10 minutes. After 5:20 hrs, cell numbers started to increase. By combining these results with the cell numbers from experiment I, we found a maximum after 3 days and decreasing cell numbers thereafter (Fig. 3).

#### Experimental design

When the experiment started on August 15^th^, 2012, plants were 6 to 9 month old. Single plants were transplanted to 6 L plastic buckets filled with a 10 cm layer of sieved sandy sediment (mesh size 1000 µm) one week before the start of the experiment. To improve growth of *Z. marina* in the new sediment, each plant was fertilized as described above. Temperature was 19°C, salinity 15–17 psu. Nine buckets were placed in each of 6 mesocosms filled with ∼600 L of seawater. In three of the six mesocosms plants were infected by using gauze bandages overgrown by *L. zosterae* (see above, Fig. 2). Plants were inoculated for different time intervals: either 0.5 hrs, 5 hrs or 50 hrs (experimental factor).Three mesocosms served as controls, in which plant leaves were wrapped with non-infected gauze bandages stored in seawater medium plates.

#### RNA extraction and reverse transcription

After incubation, a ∼4 cm leaf blade including the infection site as well as 1 cm above and below the infection site was cut and wiped with sodium hypochlorite (0.5%) to sterilize the surface. Plant tissue samples were immediately frozen in liquid nitrogen and ground with a mortar and pestle. To ensure a rapid RNA isolation, samples were taken in two time series shortly after each other.

We isolated RNA with the Invitrap Spin Plant RNA Mini kit (Stratec Molecular, Germany). Homogenized samples were kept 15–30 min in RP-lysis buffer under constant shaking. We then followed the instruction by the company. To determine the concentration of the RNA, we used a spectrophotometer (NanodropND-1000 from peQLab, Germany). RNA was transcribed to cDNA using QuantiTectReverse Transcription Kit (Qiagen, USA). Approximately 80 ng of RNA was inserted per transcription reaction. The kit contained a DNA wipe-out step to prevent gDNA contamination. As a control, we took a non-reverse transcript sample to test later in the rt-QPCR for gDNA contamination.

#### Selection of genes and primer design

Using the rt-QPCR assay, we tested 11 genes of which five genes have been previously described [40], [41]. These genes are encoding a heat shock protein and four ROS scavenging enzymes, which are known to be sensitive to biotic as well as abiotic stress. Six additional genes were identified based on homology search with known gene models from rice and *Arabidopsis* using the expressed sequence tags (EST) library database Dr. ZOMPO [39]. We chose genes that were associated with the plant pathogen defense cascade (Table 3) and made sure that these were homologous and complete when compared to other model plants using alignments. The housekeeping gene eIF4A served as reference gene for later normalization of rt-QPCR results [40]. Using the software PerlPrimer [42], primers were designed and tested for identical sequences against the EST library of *Z. marina*. Primer efficiencies (PE) were tested using a 5 fold dilution series (1∶10–1∶810) in three replicates. Efficiency E was >1.7 and R^2^ 0.87–0.99. PE was calculated according to Rasmussen *et al.* [41]:
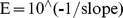



**Table 3:** *Zostera marina* genes for gene expression analysis and their predicted function.

Symbol	Gene	Predicted function	Sequence
RPPA	NB-ARC domain-containing disease resistance gene	Immune receptor	F 5′-GCATCACATCGATATCTGATTCTTT-3
			R 5′-CTGTGGTAATTTCGACCCATC-3′
EDS 5	Enhanced disease suceptibility-5	Signal molecule in SA pathway	F 5′-GATTGGGATGTGGATATGTTCTC-3′
			R 5′-GGATGTAGAAATGCCGAGGA-3′
Met-1	Metacaspase	Regulation HR	F 5′-CATTCCTTGTGCTTGAAAGTC-3′
			R 5′-ACCCTTATAGAATCCCAACGA-3′
APX*	L-ascorbate peroxidase 2 (cytosolic)	ROS regulation	F 5′-GGTGATTTCTACCAGCTTGC-3′
			R 5′-GATCCGCACCTTGGGTA-3′
CAT*	Catalase II	ROS regulation	F 5′-ACAAAATTCCGTCCGTCA-3′
			R 5′-GTCCTCAAGGAGTATTGGTCCTC-3′
GST*	Glutathione S-transferase	Detoxification	F 5′-CATGAATCCATTCGGACAAG-3′
			R 5′-CAGCAAGGTGAGTAAGGTCAG-3′
SOD*	Superoxide dismutase (mitochondrial)	ROS regulation	F 5′-ATGGGTGTGGCTTGCTTA-3′
			R 5′-ATGCATGCTCCCATACATCT-3′
HSP70**	Heat shock protein 70	Folding and unfolding of other proteins	F 5′-ACCGTCTTTGATGCGAAGC-3′
			R 5′-CAGAAAATTGCTTATCTTCTCCCTTA-3′
Prot-206	Disease resistance-responsive protein 206	Pathogenesis-related protein	F 5′-CTCTTCTAGCACGCAATTTGG-3′
			R 5′-CCGAAAATGTCTCCTTCGAG-′3
Chit	Chitinase 1-like protein	Pathogenesis-related protein	F 5′-AAACAGCCATCAGCACATGA-3′
			R 5′-GTCAGCAAATCCCTGTCCAC-3′
CYP73A	Trans-cinnamate 4-monooxygenase	Enzyme for phenol synthesis	F 5′-ATATCCACCTTGTCCATTCCC-3′
			R 5′-CTGACTTCCGATACTTGCCT-3′
eIF4A*	Eukaryotic initiation factor	Eukaryotic translation initiation factor	F 5′-TCTTTCTGCGATGCGAACAG-3′
			R 5′-TGGATGTATCGGCAGAAACG-3′

SA = salicylic acid. HR = hypersensitive response. ROS = reactive oxygen species,

* from Winters et al. 2011,

** from Bergmann et al. 20.

#### Real-time quantitative PCR-Assay (rt-QPCR)

Rt-QPCR was conducted in a StepOne Plus (Applied Biosystems, USA). In one reaction we used 10 µL SYBR green fast master mix (Applied Biosystems, USA) as provided by the company, 0.8 µL of primer reverse (final concentration 200 nM), 0.8 µL primer forward (final concentration 200 nm (0.4 µL in case of EDS-5 and Met), 4.4 µl HPLC H_2_O (4.8 µL in case of EDS-5 and Met) and 4 µL of cDNA sample, 1∶20 diluted. Cycling temperatures were 95°C 3 min (once), 95°C 20 sec, 60°C 20 sec, 72° 30 sec, 42 cycles. On each plate we used a balanced design of infected and control samples to correct for plate variation. Furthermore each plate contained the reference gene and a negative control as well as a no-template and a no-reverse transcript control (taken after genomic DNA digestion to control for genomic DNA contamination) sample.

#### Data analysis and statistics

All samples were tested in triplicate and the standard deviation of triplicates never exceeded 0.5 units of cycle threshold (Ct).

To obtain a relative measure for transcript amounts, we calculated − Δ C_t_ values (1). Fold changes in gene expression were calculated according to equation (2) and (3).
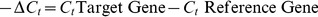
(1)

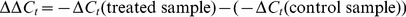
(2)

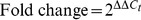
(3)


Statistical analysis was based on − ΔC_t_ values in a general linear model with − ΔC_t_ as response variable and Infection and Incubation Time (0.5, 5 or 50 hours) as independent variables. For statistical differences between incubation time levels, we conducted a Tukey post-hoc test. All statistical tests used here, were performed with the software R (R Development Core Team [43]). An overview of the results of statistical analysis is given in Table 4.

**Table 4:** Experiment II: Statistical analysis of gene expression in *Zostera marina* after inoculation with *Labyrinthula zosterae* depending on inoculation time.

	Infection				Inoculation time				Infection×incubation time				Residual
Gene	df	SS	F	p	df	SS	F	p	df	SS	F	p	SS
RPPA*	1	5.25	4.99	**<0.05**	2	16.32	7.76	**<0.02**	2	17.29	8.22	**<0.02**	35.77
EDS-5	1	11.95	1.87	ns	2	33.20	2.59	ns	2	21.50	1.68	ns	211.33
Met	1	11.83	0.99	ns	2	8.63	0.36	ns	2	12.14	0.51	ns	393.00
GST	1	184	0.89	ns	2	6505.80	15.79	**<0.01**	2	6040.60	14.66	**<0.01**	7210.60
APX	1	1.66	1.24	ns	2	8.23	3.06	ns	2	11.45	4.26	**<0.05**	49.73
CAT	1	45.84	12.79	**<0.02**	2	41.89	5.85	**<0.02**	2	60.30	8.41	**<0.02**	129.07
SOD	1	147.75	21.88	**<0.01**	2	185.26	13.71	**<0.01**	2	213.69	15.82	<0.01	270.17
HSP70	1	0.82	0.45	ns	2	0.34	2.00	ns	2	0.17	0.05	ns	70.76
Prot-206	1	0.86	0.37	ns	2	22.85	4.99	**<0.05**	2	6.55	1.43	ns	93.95
Chit	1	13.41	16.59	**<0.01**	2	19.00	11.75	**<0.01**	2	21.03	13.01	**<0.01**	33.15
CYP73A	1	120.15	21.77	**<0.01**	2	81.72	7.40	**<0.02**	2	84.70	7.67	**<0.01**	215.21

* = See Table 2 for gene descriptions.

## Results

### Experiment I: Reciprocal infection of host and endophyte with different origin

Across all experimental factors, lesion development after 24 hours indicated that infection had been successful in 187 out of 210 experimental *Zostera marina* plants (89%) inoculated with *Labyrinthula zosterae*. After 48 hours, 18% of the inoculated 3^rd^ oldest leaves were covered by lesions. Three days post inoculation (after 72 hours), lesion size had doubled to 36%. Lesion progression was slightly slower on the 2^nd^ oldest leaf, where only 24% of the leaf surface was black after 3 days. However, lesions continuously increased thereafter resulting in a lesion cover of 36% after 7, 46% after 9 and 60% after 16 days. After 10 days, black spots (6±1%) appeared on the youngest leaf (at inoculation), increasing to 10±1% after 16 days. Mortality of *Z. marina* during the experiment was very low and similar to the natural mortality in our experimental set-up. Four out of 262 plants in total (3.1%) died by the end of the experiment after 16 days (3.1%), resulting in 249 plants left.

**Figure 4:**
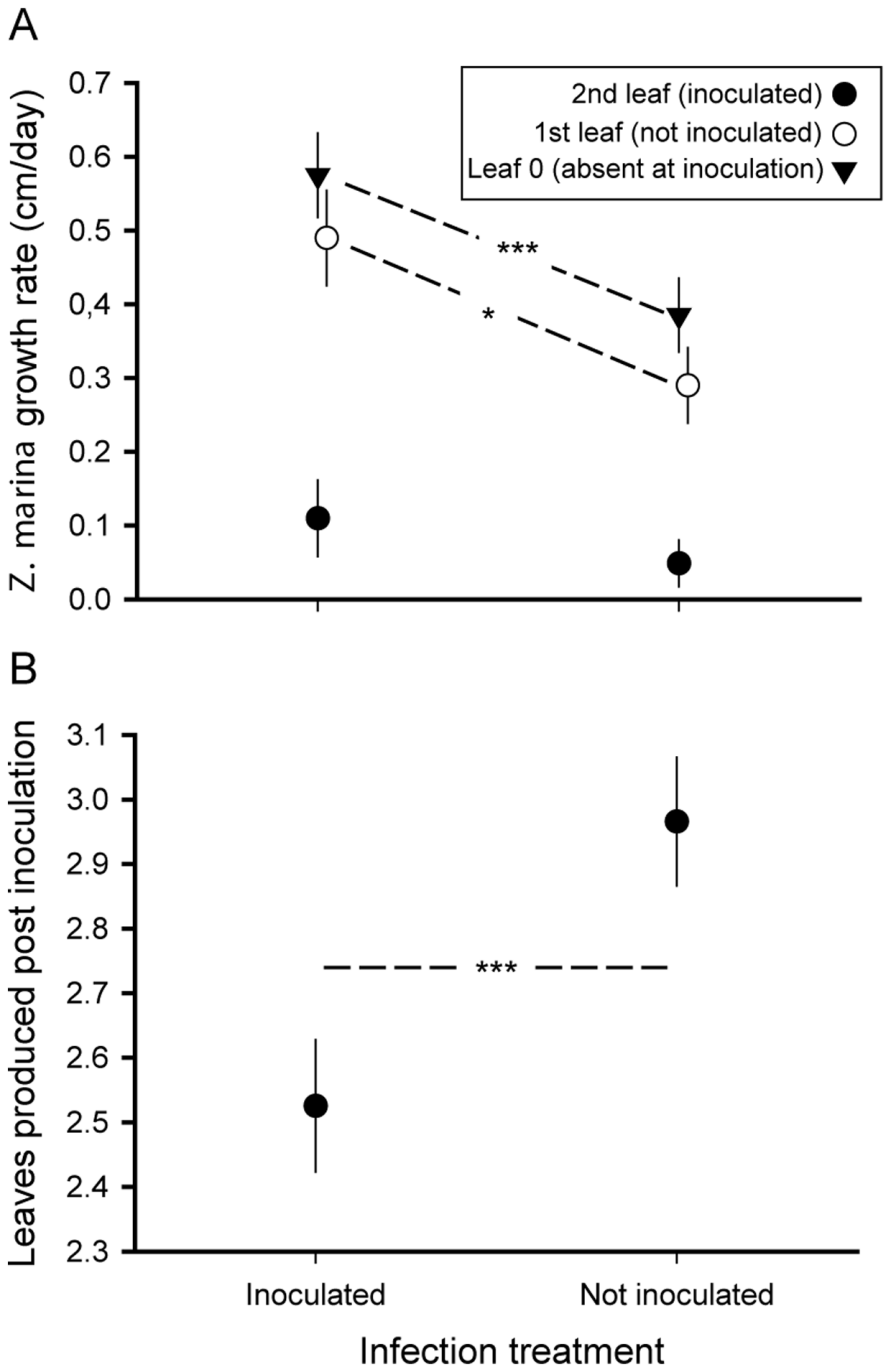
Growth (a) and leaf production (b) of *Zostera marina* leaves 2–4 weeks after experimental infection with *Labyrinthula zosterae*. 2^nd^ leaf = inoculated 2^nd^ oldest leaf of each *Zostera marina* shoot (growth measured 1^st^ to 2^nd^ week post inoculation), 1^st^ leaf = youngest leaf at inoculation, not inoculated (growth measured 1^st^ to 4^th^) week post inoculation), leaf 0 = leaf not yet present at inoculation, therefore not inoculated (growth measured 3^rd^ to 4^th^ week post inoculation). * indicates significant differences at p<0.05, *** indicates significant differences at p<0.01, ns = not significant, means with standard error bars.

Infected plants grew better than uninfected controls and showed enhanced growth of the younger leaves that were either uninfected or formed after the infection (Fig. 4a, Table 2). Furthermore infected plants produced fewer new leaves across all origins (Fig. 4b, Table 2). We found no genotype×genotype (host origin×protist origin) interactions on any of the response variables. However, there were some main effects of the factor genotype on lesion development.

**Figure 5:**
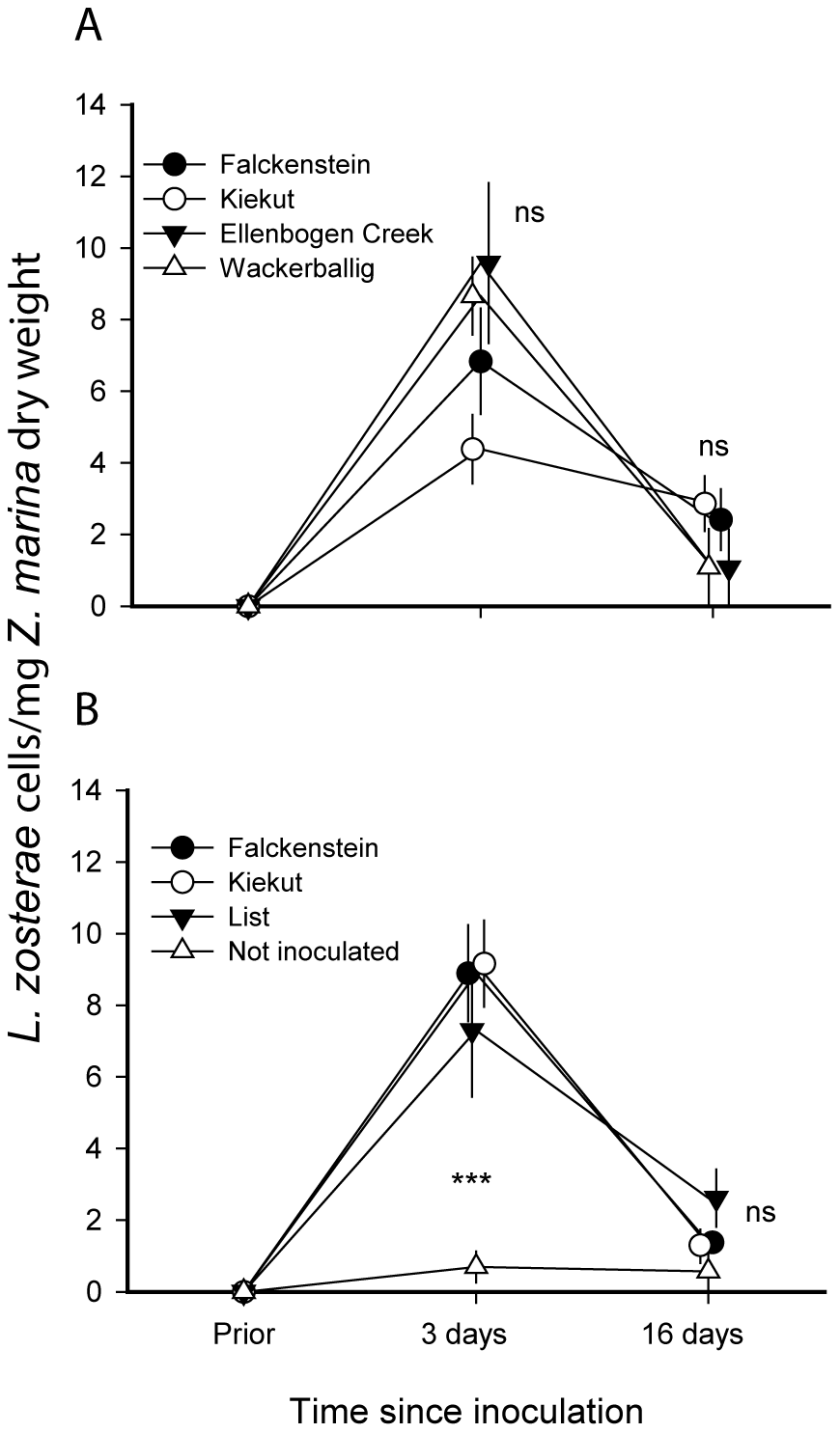
Abundance of *Labyrinthula zosterae* cells per mg *Zostera marina* leaf sample (dry weight) after experimental inoculation depending on the parental site of *Z. marina* (a) and the isolation site of *L. zosterae* (b). *** indicates significant differences at p<0.01, ns = not significant, means with standard error bars.

**Figure 6:**
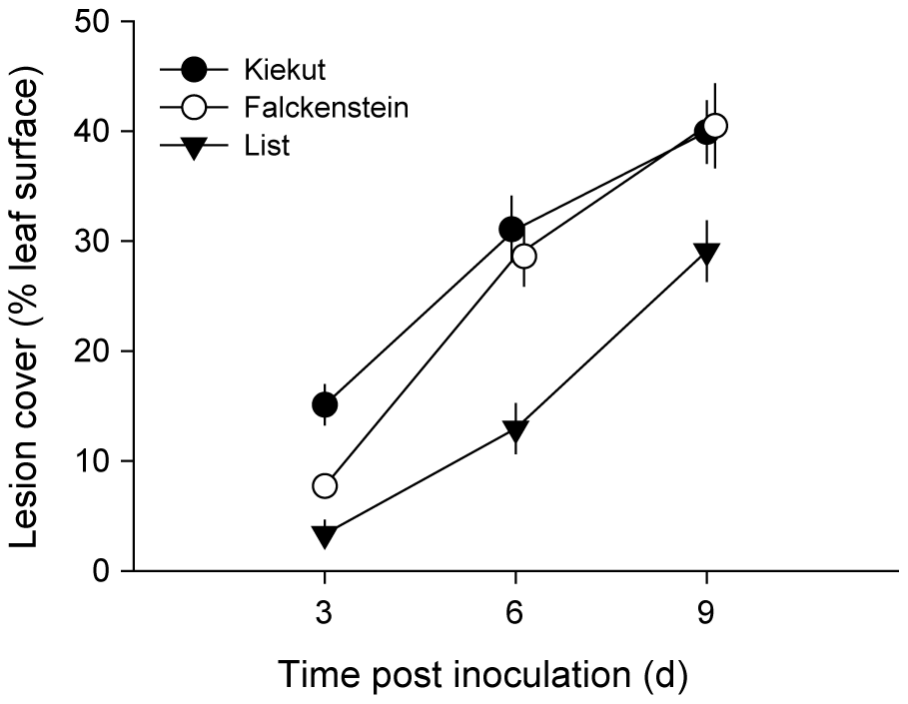
Spread of lesions on *Zostera marina* 2^nd^ oldest leaves of different origin after experimental inoculation with *Labyrinthula zosterae*, *** indicates significant differences at p<0.01, means with standard error bars.

Infected *Z. marina* plants from different origin did not differ in *L. zosterae* abundance (*L. zosterae* cells/mg *Z. marina* dry weight, Fig. 5a), leaf production or leaf growth. Origin of the *L. zosterae* culture also did not lead to significant differences in the parameters mentioned above (Fig. 5b). Seven days after infection, abundance of *L. zosterae* across all origins was reduced to low levels (Fig. 5a, b, Table 2). However, origin of the *L. zosterae* culture significantly impacted lesion progression. Infection with *L. zosterae* originating from List eelgrass beds lead to the development of significantly smaller lesions than Baltic protists (Fig. 6, Table 2).

### Experiment II: Defense gene expression in *Zostera marina*

**Figure 7:**
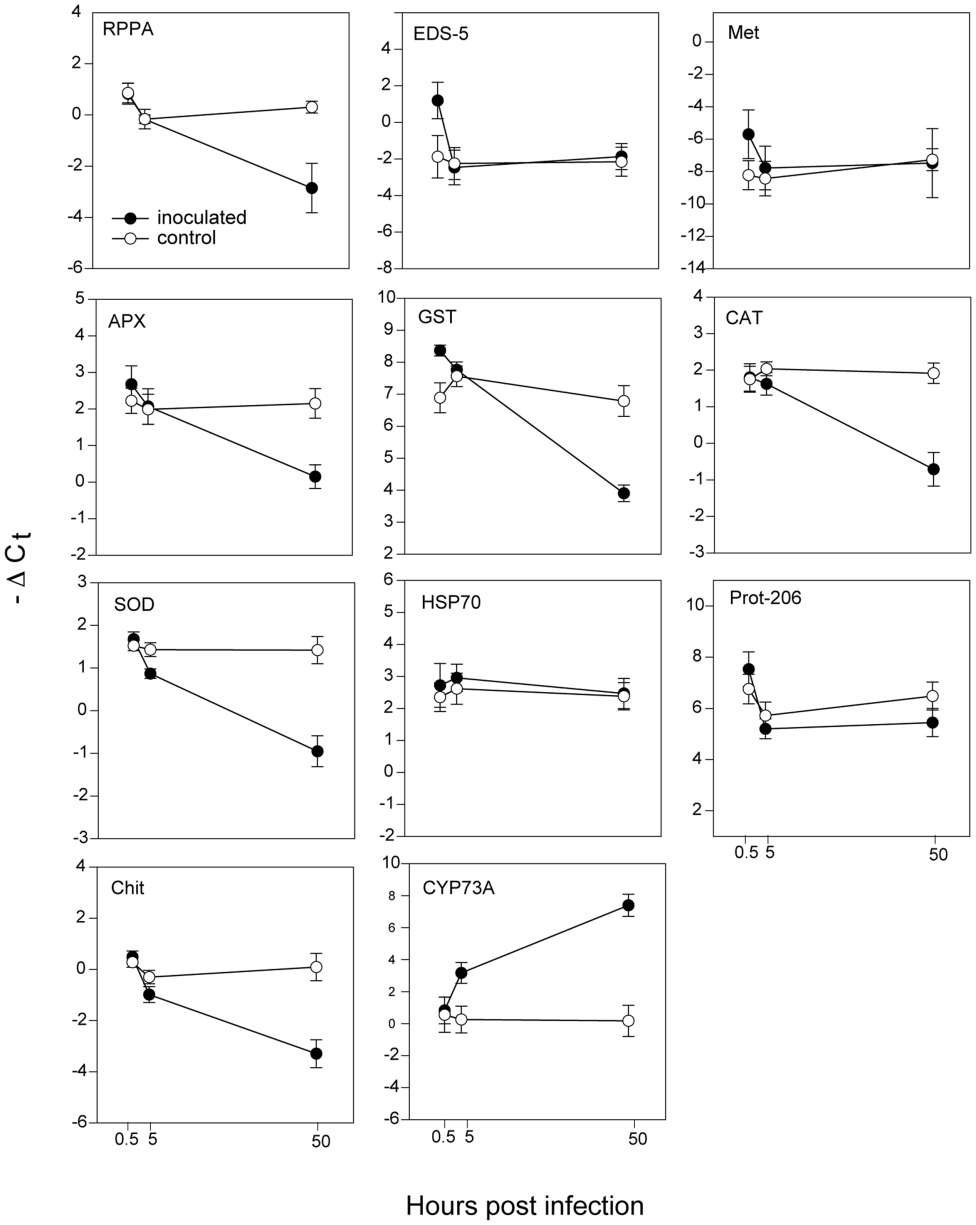
Gene expression of *Zostera marina* defense genes after experimental infection with *Labyrinthula zosterae*. I = inoculation treatment with *L. zosterae*, NI = no inoculation. Results have been normalized to *eIF4A* housekeeping gene. −ΔC_t_: log 2 scale. * indicates significant differences at p<0.5, ns = not significant. ***RPPA*** *:* NB-ARC domain-containing disease resistance receptor gene. **EDS-5**: Enhanced Disease Susceptibility 5. **Met**: Metacaspase ***APX*** *:* L-ascorbate peroxidase. **GST**: Glutathione S-transferase. **CAT**: catalase II. **SOD**: superoxide dismutase. **HSP70**: heat shock protein 70. **Prot-206**: Disease resistance-responsive protein 206. **Chit**: Chitinase. **CYP73A**: Trans-cinnamate 4-monooxygenase, means with standard error bars.

Contrary to expectations, in 6/11 defense genes, expression levels were down-regulated upon experimental infection. In relation to a housekeeping gene eIF4A, −ΔC_t_ was significantly lower in plants infected with *L. zosterae* for RPPA, APX, GST, CAT and SOD (Fig. 7, Tab. 4) with levels from 5 to 12-fold. Four genes showed no difference in expression in comparison to the housekeeping gene. In contrast, the expression of CYP73A which is involved in phenol synthesis increased almost 80-fold upon infection (Fig. 7).

## Discussion

To the best of our knowledge, we are one of the first to apply controlled infection of naïve *Z. marina* plants raised from seeds (also see [44]). Our experiments show that infection with present-day *L. zosterae* genotypes from North Sea/Baltic Sea in a non- stressful environment is not associated with the detrimental effects on *Z. marina* described for the wasting disease. Mortality levels were low and not significantly different from controls although the infectivity of the endophyte was high. Moreover, endophyte abundances inside plant tissue remained low, and decreased progressively to low levels after experimental infection, which is typical for permanent non-lethal infections [45].

The development of lesions covering significant parts of the leaf was correlated with a significant increase in growth rate of the un-inoculated younger leaves of the same shoot. Similar plant – endophyte interactions that lead to increased growth and shoot production and ultimately result in enhanced survival of the host as a consequence of infection are known from many terrestrial grass species [2], [46], [47], [48]. The mechanisms underlying this effect are for example enhanced nutrient use efficiency for nitrogen and phosphorus [3], [4], [49]. Endophyte-infected terrestrial grasses also exhibit fundamental changes in their secondary metabolites including a range of alkaloids [50], [51] and phenolic compounds [4], [52].Phenols produced by endophyte-infected grasses can not only be a reaction upon infection but for example be released through root exudates leading to an increase in P availability [52]. Along these lines, the observed ∼80 fold increase in CYP73A transcript in our study (Fig. 7) could be a direct result of host manipulation by *L. zosterae*. In addition to changes in nutrient availability, indirect beneficial effects for *Z. marina* could also be a reduction of herbivory by grazing invertebrates [53], [54], [55], which may be induced by enhanced phenolics or by infection with other microbes such as marine fungi, bacteria or viruses [31]. Furthermore, polyphenols probably control endophyte abundance by their antimicrobial function [30]. The repellent function of difference phenolic acids (e.g. caffeic acid) has previously been shown for *Z. marina* [32], [56], [57]. Moreover, phenolic compounds are also regarded as carbohydrate storage molecules in situations with nitrogen limitation [58]. Working with the subtropical seagrass *Thalassia testudinum*, Steele et al. [59] identified a correlation between infection with *Labyrinthula* sp. and the concentration of phenolic acids in plant tissue. The authors interpreted this as a consequence of over-accumulation of carbon resources in the regions above the leaf lesions (across which assimilate flow was disrupted) rather than an induced defense reaction by the plant.

The results of our transcription analysis further revealed that different layers of the host's pathogen defense were not activated: Neither R-genes (RPPA), PR-genes (Chitinase and Prot-206), genes involved in HR (Metacaspase) or signal transduction through SA (EDS-5) nor ROS scavenger genes (APX, CAT, SOD, GST) showed enhanced transcription after infection of *Z. marina* with *L. zosterae*. RPPA, Chitinase and all measured ROS scavenger genes even showed a significant 5–15-fold down-regulation (Table 4). Moreover, expression of the general stress indicator gene HSP70 was not changed due to infection (Fig. 7). This indicates that the plants were not generally stressed upon the experimental inoculation procedure. This is the first report of any marine plant that describes such immune modulation of the host defense by a potential parasite, here a protist.

Many pathogens have evolved mechanisms to manipulate host response by suppressing defense reaction e.g. through effector proteins [34], [60], [61]. One example, where several pathogenesis related (PR) genes and other genes from the defense cascade are down-regulated after infection with *Phytophthora citricola*, is *Fagus sylvatica* [62]. The author concluded that *P. citricola* escaped recognition by the host, probably by repressing it. How such an effector might work, has recently been shown by de Jonge et al. [63]. The LysM effector Ecp6 in *Cladosporium fulvum* binds Chitin and prevents thereby a Chitin-triggered host response. Comparably, *L. zosterae* might release a related effector that oppresses immune induction in *Z. marina*. In our study, the tested resistance-gene immune receptor (RPPA, involved in recognition of pathogens), as well as the pathogenesis-related proteins (Chitinase and Prot-206 from the base of the signal cascade) are non-differential or lower expressed in infected plants, supporting this theory.

Another indication that the endophyte manipulates the defense reaction of *Z. marina* is the down regulation of ROS scavenging genes (SOD, CAT, APX, GST).ROS is a crucial signal for HR and other pathogenesis related defense mechanisms and does therefore play an important role in plant-pathogen interaction [64]. The observed down regulation of ROS scavenging genes (SOD, CAT, APX and GST) in *L. zosterae* infected eelgrass, especially SOD which catalyzes the dismutation of superoxide (O_2_−) to oxygen and hydrogen peroxide might imply that the eelgrass does not recognize *L. zosterae*. Robb *et al.* [65] observed a comparable down regulation of host antioxidant enzymes in the tolerant interaction between the tomato strain *Lycopersicon esculentum* and the pathogen *Verticillium dahliae*, concluding that no oxidative burst occurs in these plants. Alternatively, the down-regulation of antioxidant enzymes could also result in an accumulation of reactive oxygen species (ROS) resulting in damage of plasma- and compartment-membranes and macromolecules [66]. In consequence, plant cell exploitation and symplastic movement of *L. zosterae* might be facilitated through non-functional cell components [67].

Although *L. zosterae* has no severe impact on *Z. marina* in our study area today, it is very well possible that this may change as shown in many other examples of host-microbe associations [68], [69]. Survival of eelgrass strongly depends on the leaf turn-over rate: As long as new leaves grow faster than old leaves decay, the survival is assured. But if growth will be reduced through abiotic or biotic stressors, leaf mortality may outbalance leaf growth. Predominant general stressors for *Z. marina* are increasing water temperatures in the face of global climate change and reduced light availability caused by eutrophication [16], [17], [22], [41], [70]. Potentially, these stressors could alter the actually non-virulent relationship between eelgrass and its endophyte towards pathogenicity.

We can conclude that under our non-stressful experimental conditions, *L. zosterae* infection in the study region is not associated with the detrimental effects on *Z. marina* described for the wasting disease. Although infectiousness of the endophyte was high, we found no evidence that *Z. marina* is negatively impacted by *L. zosterae* infection. Instead *Z. marina* seemed to profit through enhanced leaf growth and kept endophyte abundance low possibly as a consequence of high concentrations of phenolic acids. We hypothesize that under adverse conditions (e.g. high water temperatures, low light availability) imposing stress on *Z. marina*, the protist-plant relationship may become pathogenic.
